# Mitochondrial abnormalities contribute to muscle weakness in a *Dnajb6* deficient zebrafish model

**DOI:** 10.1093/hmg/ddae061

**Published:** 2024-04-15

**Authors:** Emily A McKaige, Clara Lee, Vanessa Calcinotto, Saveen Giri, Simon Crawford, Meagan J McGrath, Georg Ramm, Robert J Bryson-Richardson

**Affiliations:** School of Biological Sciences Monash University, 25 Rainforest Walk, Clayton, VIC 3800, Australia; School of Biological Sciences Monash University, 25 Rainforest Walk, Clayton, VIC 3800, Australia; School of Biological Sciences Monash University, 25 Rainforest Walk, Clayton, VIC 3800, Australia; Department of Biochemistry and Molecular Biology, Biomedicine Discovery Institute, Monash University, 23 Innovation Walk, Clayton, VIC 3800, Australia; Monash Ramaciotti Centre for Cryo Electron Microscopy, Monash University, 15 Innovation Walk, Clayton, VIC 3800, Australia; Department of Biochemistry and Molecular Biology, Biomedicine Discovery Institute, Monash University, 23 Innovation Walk, Clayton, VIC 3800, Australia; Department of Biochemistry and Molecular Biology, Biomedicine Discovery Institute, Monash University, 23 Innovation Walk, Clayton, VIC 3800, Australia; Monash Ramaciotti Centre for Cryo Electron Microscopy, Monash University, 15 Innovation Walk, Clayton, VIC 3800, Australia; School of Biological Sciences Monash University, 25 Rainforest Walk, Clayton, VIC 3800, Australia

**Keywords:** dnajb6, myopathy, mitochondria, zebrafish, disease model

## Abstract

Mutations in *DNAJB6* are a well-established cause of limb girdle muscular dystrophy type D1 (LGMD D1). Patients with LGMD D1 develop progressive muscle weakness with histology showing fibre damage, autophagic vacuoles, and aggregates. Whilst there are many reports of LGMD D1 patients, the role of DNAJB6 in the muscle is still unclear. In this study, we developed a loss of function zebrafish model in order to investigate the role of Dnajb6. Using a double *dnajb6a* and *dnajb6b* mutant model, we show that loss of Dnajb6 leads to a late onset muscle weakness. Interestingly, we find that adult fish lacking Dnajb6 do not have autophagy or myofibril defects, however, they do show mitochondrial changes and damage. This study demonstrates that loss of Dnajb6 causes mitochondrial defects and suggests that this contributes to muscle weakness in LGMD D1. These findings expand our knowledge of the role of Dnajb6 in the muscle and provides a model to screen novel therapies for LGMD D1.

## Introduction

Mutations in *DNAJB6* are a known cause of limb girdle muscular dystrophy D1 (LGMD D1) [1–5]. Patients show variability in disease severity, typically showing signs of mild muscle weakness during early adulthood and then losing walking ability after 20 years, which significantly impacts their quality of life [6, 7]. Typically, cardiac and respiratory defects are not observed however, multiple patients have been identified with difficulty swallowing [8, 9]. The majority of affected individuals have dominant missense mutations, with the mutant protein thought to exert a dominant negative effect on the chaperone activity of wildtype DNAJB6 [4]. Recently, recessive cases were also documented, where reduced DNAB6a protein expression occurs [10], expanding the spectrum of *DNAJB6* mutations. Since the first reported case of LGMD D1 over 10 years ago a number of cellular and animal models have been utilised to better understand the function of DNAJB6 [10–13]. Despite this, the underlying disease mechanism of LGMD D1 and the function of DNAJB6 in the muscle is still not fully resolved. This study seeks to further explore the requirement for DNAJB6 in the muscle and improve our understanding of how its disrupted function leads to myopathy.

DNAJB6 is comprised of three key domains, the J domain, the G/F domain and the C terminal domain. LGMD D1 mutations are typically found within the G/F domain, however, recently, mutations in the J and C terminal domains have also been documented [10, 14]. *DNAJB6* is ubiquitously expressed and has two isoforms, DNAJB6a which is found in the nucleus and a shorter isoform, DNAJB6b, which is found in the cytoplasm and is localised to the Z-disk in muscle [15, 16]. Although initial studies reported only the cytoplasmic isoform as disease causing, it is now known that mutations specifically affecting the nuclear isoform can also cause LGMD D1 [10, 11]. The exact cellular function of these isoforms is still unresolved.

DNAJB6 is a highly conserved protein which belongs to the Hsp40/DnaJ protein family. The DnaJ family of co-chaperones are important for stabilising and providing specificity for the interaction between Hsp70 and unfolded or misfolded target proteins [17]. The formation of this trimeric complex relies on DnaJ proteins stimulating the ATPase activity of Hsp70 through their J domain [18]. Together HSP70 and DNAJB6 form a chaperone complex with aggregation suppression properties [19]. Recent work has shown that mutation of DNAJB6 does not affect its aggregation properties, however, does result in unregulated activation and depletion of Hsp70, leading to inefficient protein quality control [20]. This is supported by another study showing that inhibition of the interaction between DNAJB6 and Hsp70 improves muscle appearance and strength in *DNAJB6* mutant mice [13]. Taken together, this suggests that dominant mutations in DNAJB6 affect Hsp70 function, which disrupts protein homeostasis and leads to myopathic changes.

Another function of DNAJB6 in the muscle could be related to the macroautophagy protein turnover pathway. This is based upon the interaction of DNAJB6 with the chaperone assisted selective autophagy (CASA) proteins BAG3 and HSPB8 [11], the increased aggregation of proteins, rimmed vacuoles, and accumulation of LC3-II and p62/SQSTM1 seen in patient muscle [10, 21, 22]. Previous work using cell, mouse, and fly models have identified that these aggregates contain sarcomeric proteins (DES, ACTN, and FHL1), chaperones (CRYAB, HSPA8, HSPB8), and RNA-binding proteins (TDP-43, hnRNPA1, and hnRNPA2) [11–14, 16, 23, 24]. Interestingly, some of these proteins have ties to the autophagy pathway, for instance, *in vitro* studies have shown that the RNA-binding protein TDP-43 targets the autophagic mRNAs *ATG7*, *RPTOR*, and *DCTN1* [25, 26]. A study using a transgenic *DNAJB6* mutant mouse model showed that treatment with LiCl reduced disease severity [12]. Treated mutant mice had increased muscle strength, larger muscle mass, and decreased variability in muscle fibre size. These improvements were attributed to inhibition of GSK3β and subsequent activation of downstream myogenic signalling pathways. However, LiCl is also an activator of autophagy [27] and it is possible these improvements are due to increased autophagy. Questions remain as to whether DNAJB6 is involved in the autophagy pathway.

The aim of this study is to better understand the role of DNAJB6 in muscle. In zebrafish there are two orthologs of *DNAJB6*, *dnajb6a* and *dnajb6b,* and each gene has two isoforms. Using CRISPR-Cas9 we created a double mutant strain. We demonstrate that loss of Dnajb6 results in a late onset muscle weakness, and analysis of adult double mutant fish revealed no signs of aggregation, disrupted autophagy or myofibril disorganisation. Interestingly, we show that double mutants display mitochondrial abnormalities including decreased cristae content. Our findings suggest that mitochondrial changes may contribute to muscle weakness in LGMD D1.

## Results

### *dnajb6a* and *dnajb6b* are expressed during embryonic development, and fish mutant for *dnajb6a* and *dnajb6b* show nonsense mediated decay of both transcripts

**Figure 1:**
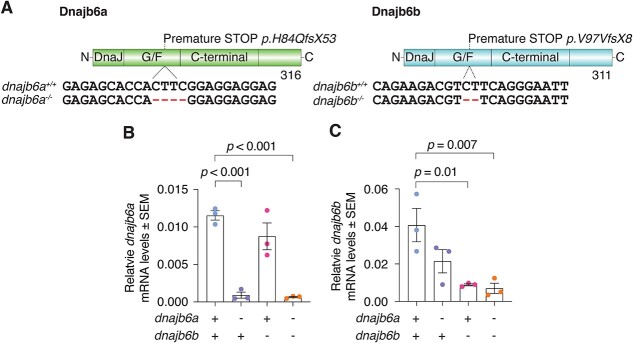
Generation of a *dnajb6* loss of function mutant. (A) Zebrafish mutant lines for *dnajb6a* and *dnajb6b*. Both mutations result in premature STOP codons. (B) qRT-PCR analysis shows a significant loss of *dnajb6a* transcript in single and double mutants compared to their wildtype siblings. (C) Significant decrease in *dnajb6b* levels in single and double mutants relative to wildtype levels at 3 mpf. Both mutations appear to result in nonsense mediated decay. Analysis was completed in triplicate and error bars represent SEM. Values are relative to the mean of *lsm12b* and *ef1α* values. Genotypes were compared using a one-way ANOVA and two-way Dunnett multiple comparisons test.

To explore the role of DNAJB6 in muscle we utilised zebrafish as a model system. In order to determine the stage of embryonic development the zebrafish *DNAJB6* orthologues *dnajb6a* and *djajb6b* are expressed, RT-PCR was performed on wildtype cDNA samples from 1–6 days post fertilisation (dpf). Expression of *dnajb6a* and *dnajb6b* was detected at low levels across all embryonic stages (Supp. Fig. 1). We then used CRISPR-Cas9 genome editing to create a double *dnajb6* mutant model. For both *dnajb6a* and *dnajb6b* deletions were generated in the G/F domain that lead to premature STOP codons (Fig. 1A). These two strains were then crossed together to create a double mutant *dnajb6* line. At 6 dpf *dnajb6* mutants were phenotypically indistinguishable from their siblings (Supp. Fig. 2). Using qRT-PCR we assessed levels of *dnajb6a* and *dnajb6b* transcripts in our mutants, to determine if the transcripts were degraded and if there was any compensatory increase in expression of the paralogue. Double mutant fish had a significant loss of both transcripts compared to their wildtype siblings with *dnajb6a* and *dnajb6b* mutants each showing loss of their respective transcripts, presumably due to nonsense mediated decay (Fig. 1B and C). Therefore, the *dnajb6* double mutant is likely a loss of function model. Loss of either *dnajb6a* or *dnajb6b* transcripts did not result in increased expression of the other gene.

### *dnajb6a^−/−^;dnajb6b^−/−^* fish do not show defects in muscle structure

**Figure 2:**
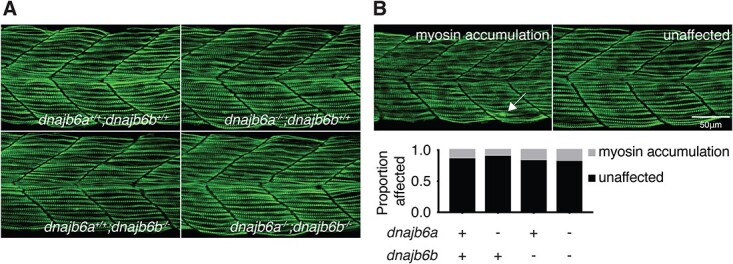
Double *dnajb6* mutant zebrafish do not show signs of myopathy during early development. (A) Immunofluorescence staining of methyl cellulose treated *dnajb6* mutants and their siblings with anti-myosin at 1 dpf. Larvae were incubated in methyl cellulose for 1 h prior to fixation. Maximum projection confocal images were taken laterally of the trunk above the egg yolk extension. (B) Images show examples of the phenotype scored. Quantification and analysis of the fibre damage phenotype showed no significant differences between genotypes (*P* > 0.05, binomial test). Analysis was completed with *n* ≥ 28 per genotype.

As individuals with *DNAJB6* myopathy exhibit muscle damage we first analysed myofibril structure in our model. In order to place the muscle under load embryos were incubated in a viscous methyl cellulose solution, which has previously been shown to induce fibre damage in a zebrafish myofibrillar myopathy model [28]. We then performed immunostaining at 1 dpf with a myosin antibody to label the thick filament. At this early timepoint neither single nor double *dnajb6* mutant fish show signs of compromised fibre integrity compared to wild type siblings (Fig. 2). We then assessed Z-disk structure using an α-actinin antibody at 2 dpf. There were no signs of Z-disk damage in either single or double mutant fish (Supp. Fig. 3). Together these results indicate that loss of *dnajb6* does not lead to changes in muscle structure during early development.

### Loss of *dnajb6a* and *dnajb6b* results in late onset muscle weakness and decreased survival.

**Figure 3:**
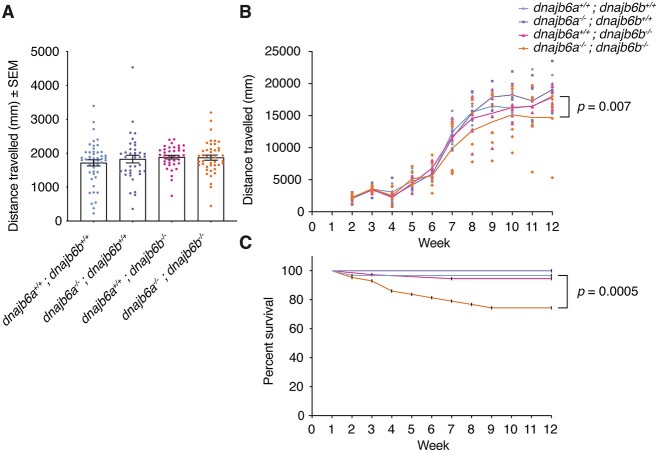
Functional analysis reveals late onset muscle weakness in double *dnajb6* mutants. (A) The distance travelled was not significantly different between genotypes at 6 dpf. The swimming of *dnajb6* fish was recorded for a period of 10 min and each dot represents an individual fish. Analysis was completed in triplicate with *n* ≥ 38 per genotype. Error bars show standard error of the mean (SEM). Genotypes were compared using a linear model. (B) Double mutant fish show a significant decline in motor function over time (*P* = 0.007). Fish were genotyped prior to the start of the experiment and their movement was recorded weekly from 2 weeks post fertilisation (wpf) to 12 wpf. Each dot represents the average swimming distance of one group, with six groups per genotype. Analysis was completed with *n* ≥ 34 per genotype from week 2 onwards and a mixed linear model. (C) Survival of double mutant fish was significantly less than wildtype fish (*P* = 0.0005). Analysis was completed with *n* ≥ 29 per genotype from week 1 onwards. Kaplan–Meier survival curves were compared using the mantel-cox log rank test.

Muscle function was next evaluated using swimming assays. At 6 dpf, no difference in the distance travelled by double *dnajb6* mutants was observed when compared to wild type (Fig. 3A). Given *DNAJB6* patients typically begin to show signs of muscle weakness during adulthood, *dnajb6* zebrafish were examined at later stages. Interestingly, over time double *dnajb6* mutants, but not single *dnajb6a* or *dnajb6b* mutants, show a progressive decline in motor function (Fig. 3B, *P* = 0.007). This indicates a late onset of muscle weakness in our model. A significant decline in the survival of double mutant animals was observed with age, compared to their wildtype, or single mutant siblings (Fig. 3C, *P* = 0.0005).

### *dnajb6a;dnajb6b* mutants only show autophagy defects during early development

**Figure 4:**
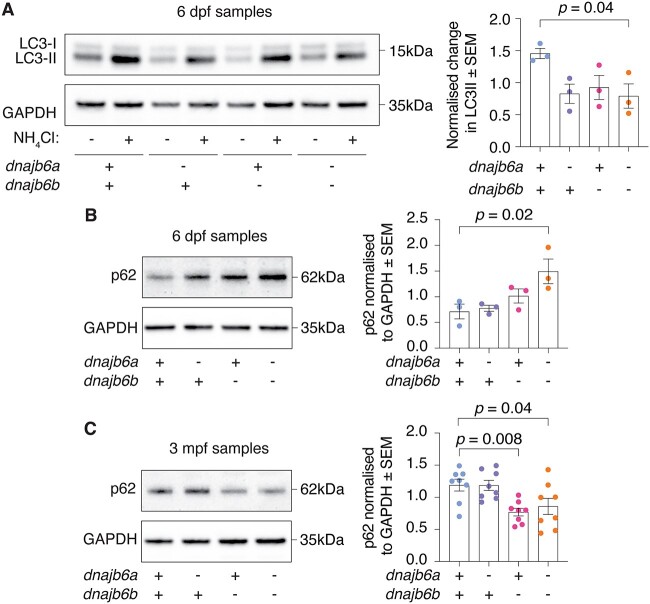
Analysis of autophagy markers reveals early defect in double mutants. (A) Double mutant fish show a significant reduction in autophagic flux activity at 6 dpf based on difference in LC3-II levels between NH_4_Cl treated and untreated zebrafish. Experiment was completed in triplicate. (B) Western blot at 6 dpf shows a significant accumulation of p62 in double mutants. Error bars represent SEM for three biological replicates. (C) At 3 mpf *dnajb6b* single mutants and double mutants show a significant reduction in p62 levels compared to wildtype. Error bars represent SEM for eight fish across 3 biological replicates. GAPDH was used as a loading control and P62 values are normalised to GAPDH. Datasets were analysed with a one-way ANOVA and a two-way Dunnett multiple comparisons test.

To determine the underlying cause of muscle weakness we considered the role of autophagy, as mutation of *DNAJB6* has previously been associated with autophagic defects [11, 21] and defects in autophagy are a known cause of skeletal myopathy [28, 29]. To assess if double mutants had an autophagy defect, we analysed LC3-II levels in untreated and NH_4_Cl treated larvae to measure autophagic flux (Fig. 4A). Treatment of larvae with NH_4_Cl prevents autophagolysosome formation and suppresses the activity of lysosomal hydrolases, blocking the autophagy pathway, and causing a build-up of LC3-II. Analysis of LC3-II build-up, relative to the basal levels found in untreated samples, gives an indication of the activity of the autophagy pathway, as previously described [28]. Double *dnajb6* mutants showed a significant reduction in autophagic activity, with decreased LC3-II flux compared to their wildtype siblings at 6 dpf (*P* = 0.04). The differences between the control and single mutants were not significant. To confirm this autophagy defect, accumulation of the autophagy receptor SQSTM1/p62 was examined, revealing a significant increase in double mutants at 6 dpf (Fig. 4B, *P* = 0.02). Together, these results demonstrate that loss of Dnajb6a and Dnajb6b at 6 dpf results in an autophagy defect. However, at this timepoint we did not observe a functional deficit, indicating that this autophagy defect is not sufficient to impair muscle function. However, impaired muscle function was noted in double mutants as they aged to adulthood, so autophagy was examined by assessing levels of p62 at 3 mpf (Fig. 4C). Interestingly, a significant decrease in p62 levels was detected in the *dnajb6b* single mutant and double mutant fish (Fig. 4B, *P* = 0.008 and *P* = 0.04 respectively), suggesting autophagy changes also occur at this age, but may differ from effects in younger fish.

### Double *dnajb6* mutants develop mitochondrial abnormalities in the absence of myofibril damage or autophagy defects

**Figure 5:**
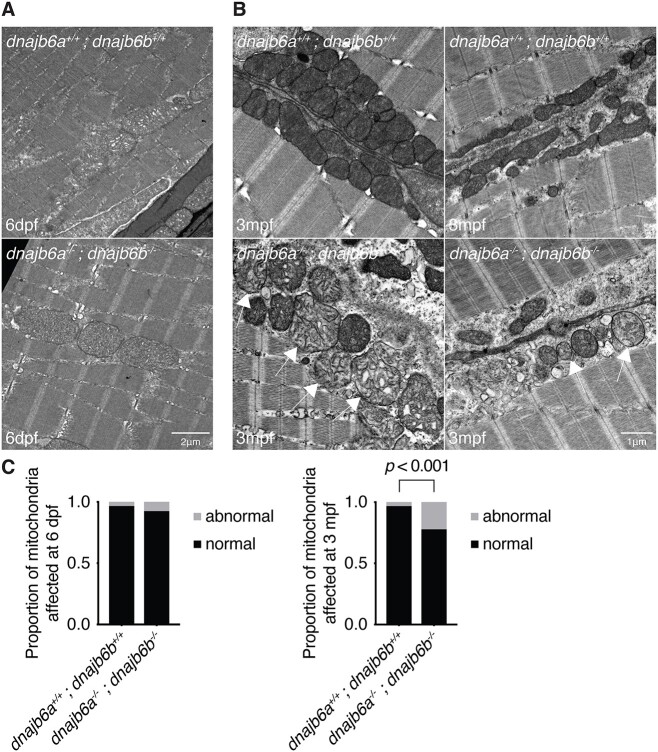
Double *dnajb6* mutants show mitochondrial abnormalities. (A) Electron microscopy of double mutant and wildtype muscle at 6 dpf shows no visible difference between double *dnajb6* mutants and wildtype. (B) Ultrastructure of 3 mpf double mutant and wildtype muscle reveals mitochondria with abnormal cristae arrangement (arrow). (C) Quantification of mitochondrial abnormalities at 6 dpf and 3 mpf. At 6 dpf there was no sign of mitochondrial abnormalities in double mutants. However, at 3 mpf there was a significant increase in the proportion of abnormal mitochondria in double mutants compared to wildtype (*P* < 0.001, based on a binomial test). Analysis at both timepoints was completed blinded with two biological replicates and *n* = 4 per genotype.

To further investigate the underlying reasons for the reduction in motor activity, EM analysis was performed at 6 dpf and 3 mpf, with scoring of phenotypes whilst blinded to genotype. Surprisingly, at both timepoints there were no signs of Z-disk disruption, aggregation or autophagic vacuoles in double *dnajb6* mutants, features that are commonly seen in *DNAJB6* myopathy patients [11, 14]. This is in line with our p62 results on muscle samples from adult fish, which suggested double mutants do not have an autophagy defect. At 6 dpf we saw no significant differences in the prevalence of abnormal mitochondria in double mutants compared to wildtype (Fig. 5A and C). Examples of abnormal mitochondria at 6 dpf are shown in Supplementary Fig. 4. Interestingly, a significant decrease in mitochondrial size was observed in the muscle of double mutants at 6 dpf (Supp. Fig. 5B). At 3 mpf, our analysis showed that double mutants have a significantly higher proportion of abnormal mitochondria with diffuse cristae (Fig. 5B and C). Importantly, this matches ultrastructure findings in LGMD D1 patients [10, 30]. Therefore, these results suggest that mitochondrial abnormalities, rather than structural or autophagic defects, may underlie the observed reduction in muscle function.

### Double *dnajb6* mutants display signs of mitochondrial damage

**Figure 6:**
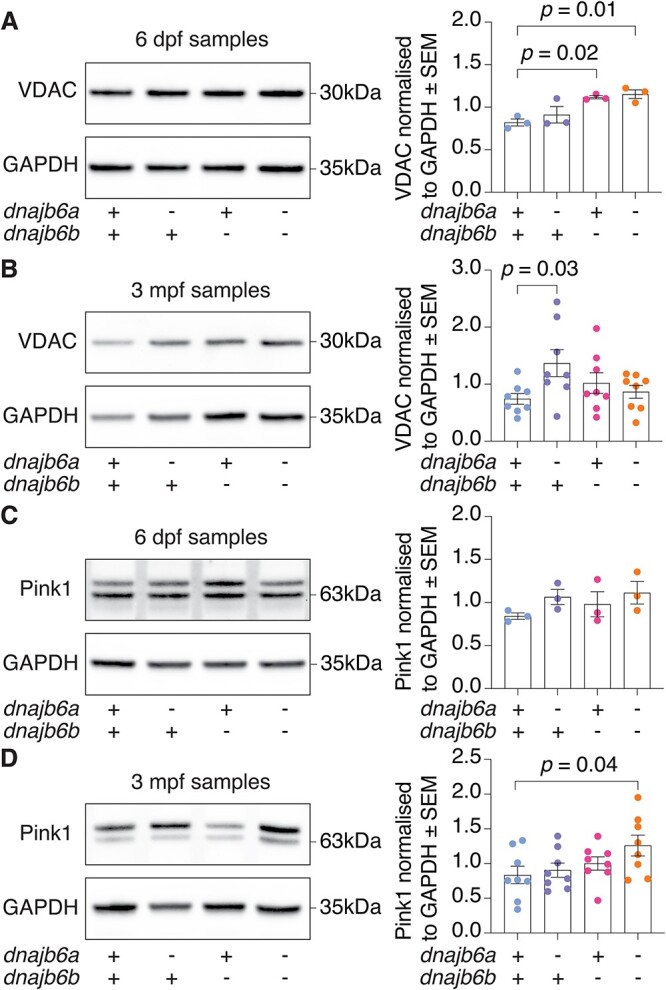
Western blot analysis shows *dnajb6* double mutants have an increase in Pink1 levels indicating mitochondrial damage. (A) VDAC protein levels were increased at 6 dpf in *dnajb6b* mutants and double mutants. (B) At 3 mpf VDAC protein levels were significantly increased in *dnajb6a* single mutants but not in double mutants. (C) Western blot of Pink1 at 6 dpf shows no significant differences between genotypes. (D) Double mutants show significantly higher levels of Pink1 compared to their wildtype siblings at 3 mpf. Values are normalised to wildtype and GAPDH was used as a loading control. Error bars represent SEM for three biological replicates at 6 dpf and eight samples from 3 biological replicates at 3 mpf. Analysis was completed using a one-way ANOVA and two-way Dunnett multiple comparisons test.

To further examine the mitochondrial phenotype observed in *dnajb6* mutants, levels of the outer mitochondrial membrane marker VDAC were examined. At 6 dpf we discovered a significant increase in VDAC levels in double *dnajb6* mutants compared to their wildtype siblings (Fig. 6A, *P* = 0.01). VDACs encode voltage-gated pore proteins found in the outer mitochondrial membrane and VDAC levels are sometimes used as a proxy for mitochondrial numbers. Our electron microscopy analysis, which directly assessed mitochondrial number, showed no significant difference in double mutants at 6 dpf, and even a small reduction in size (Supp. Fig. 5A and B). Therefore, we believe the increase in VDAC levels indicates a disruption to mitochondrial homeostasis, rather than a change in mitochondrial number. At 3 mpf we found a significant increase in VDAC levels in *dnajb6a* single mutants (Fig. 6B, *P* = 0.03), however, not in double *dnajb6* mutants (Fig. 6B, *P* = 0.92). This is in line with our electron microscopy analysis, which showed no change in mitochondrial size or number in double mutants at 3 mpf (Supp. Fig. 5D). We next analysed levels of Pink1 which is degraded in the absence of mitochondrial damage, but subsequently accumulates on the outer mitochondrial membrane when damaged mitochondria are targeted for degradation via autophagy (mitophagy) [31]. Our analysis revealed two Pink1 bands, with the larger band showing phosphorylated Pink1 as previously described [32]. Both bands were used for quantification of Pink1 levels. There was no significant difference in the levels of Pink1 between genotypes at 6 dpf (Fig. 6A, *P* = 0.26). However, at 3 mpf double mutants showed a significant increase in Pink1 levels (Fig. 6B, *P* = 0.04). Given that Pink1 accumulates on the outer membrane of damaged mitochondria to induce mitophagy [33], increased Pink1 levels are consistent with signs of mitochondrial damage in double mutants.

### *dnajb6a;dnajb6b* mutants show changes to mitochondrial metabolism

**Figure 7:**
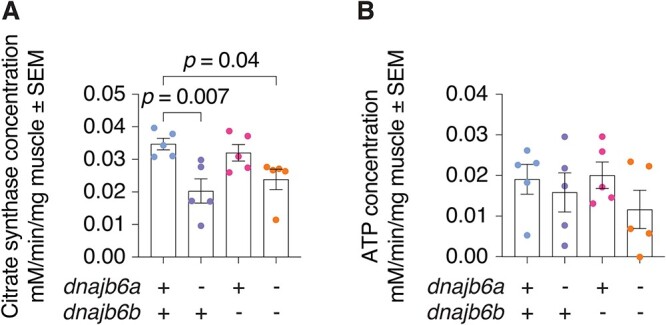
Mitochondrial cristae content is decreased in double *dnajb6* mutants. (A) Activity levels of citrate synthase are decreased in *dnajb6a and dnajb6a;dnajb6b* mutants at 3 mpf compared to wildtype siblings. (B) Analysis of ATP levels suggests no significant differences between genotypes at 3 mpf. Graph shows values normalised to time and mg of muscle tissue. Error bars represent SEM for five samples. Analysis was completed using a one-way ANOVA and two-way Dunnett multiple comparisons test.

Given the changes to mitochondrial appearance and markers in adult fish lacking Dnajb6 we next examined mitochondrial abundance and function using citrate synthase and ATP measurements respectively at 3 mpf. These markers are part of different stages of cellular respiration: citrate synthase localises to the mitochondrial matrix, found between cristae, and is the first enzyme in the citric acid cycle which is involved in the metabolism of carbohydrates, amino acids and fatty acids. On the other hand, ATP is primarily produced via oxidative phosphorylation through the electron transport chain at the cristae of the inner mitochondrial membrane. Analysis of ATP levels revealed highly variable results with no significant difference between genotypes (Fig. 7B). Interestingly, a significant decrease in citrate synthase activity occurs in single *dnajb6a* mutants (*P* = 0.007) and double *dnajb6* mutants (Fig. 7A, *P* = 0.04). Given that double mutants did not show any changes to mitochondrial size or number (Supp. Fig. 3C and D), this difference in citrate synthase activity is unlikely due to alteration of mitochondrial mass. Given that our EM imaging did identify the accumulation of abnormal mitochondria in double mutants including marked changes to cristae architecture, we propose these ultrastructural defects impact mitochondrial metabolic processes in muscle which impacts motor function.

## Discussion

Numerous studies have identified *DNAJB6* mutations as a cause of skeletal myopathy however, the function of *DNAJB6* in the muscle is still unclear [3, 5, 6, 11, 22]. This study focused on uncovering the role of *DNAJB6* in muscle using zebrafish as a model system. With CRISPR-Cas9 technology we created a double *dnajb6* mutant that showed loss of both *dnajb6* transcripts via RT-PCR. Immunofluorescence staining of muscle fibres revealed double mutants with normal development of muscle structure at 1 dpf. And at 6 dpf double mutants showed motor activity that was comparable to their wildtype siblings. Our finding that loss of *dnajb6a* or *dnajb6b* does not result in a decrease in motor activity is in contrast to a previous morpholino study [34]. However, this discrepancy could be due to maternal deposition of Dnajb6a and Dnajb6b transcripts, both of which have been identified at the zygote stage [35]. In contrast, we showed that double *dnajb6* mutants had a progressive decrease in their muscle function with age. This is consistent with *DNAJB6* mouse models, and LGMD D1 patient data, where over 70% of cases do not show muscle weakness until early adulthood [10, 12, 13, 36]. Interestingly, this shows that loss of wildtype DNAJB6 function, rather than a dominant toxic effect, is sufficient to induce myopathy symptoms. We also observed a small but significant decrease in survival in double mutants, which matches that seen in transgenic *DNAJB6* mutant mice [16]. Interestingly, loss of *Dnajb6* in mice was previously shown to result in embryonic lethality due to aggregation of keratin intermediate filaments and failure of chorioallantoic attachment [37, 38]. Whilst *DNAJB6* patients also show aggregation of KRT18 [11], most patients have dominant missense mutations and the recently discovered recessive case of *DNAJB6* myopathy showed loss of the nuclear DNAJB6 isoform but no significant change to levels of the cytoplasmic DNAJB6 isoform [10], therefore patients with a complete loss of DNAJB6 have not been identified, potentially due to embryonic lethality. Therefore, an advantage of the zebrafish, which does not have this attachment, is that loss of Dnajb6 and its effect on the muscle can be studied.

Previous studies have considered the dominant toxic effect of DNAJB6 in the muscle, identifying uninhibited Hsp70 activation as a cause of aberrant protein quality control and myofibril structural damage. In this study, we investigated possible functions of wildtype DNAJB6 in the muscle. We first considered whether loss of Dnajb6 caused an impairment in autophagy, as previous work suggested DNAJB6 could have a role in this pathway [11]. Interestingly, western blot analysis of LC3-II and p62 revealed a defect in autophagy during early development however, not at adult stages. This suggests that loss of both Dnajb6 proteins leads to an initial defect in autophagy that does not persist over time. Our results suggest that dysfunctional autophagy does not contribute to muscle weakness in double mutants at adult stages. This contrasts with patient data and a *DNAJB6a* frameshift mouse model, which both showed an accumulation of p62 and LC3-II aggregates in adult myofibers, with mutant mice also showing an increase in LC3-II protein levels [10]. This difference is interesting, as it suggests that other mechanisms contribute to the onset of *DNAJB6* LGMD.

To explore the underlying reason behind muscle weakness we performed ultrastructure analysis on adult muscle tissue. The features typically identified in *DNAJB6* myopathy patients include autophagic vacuoles, aggregation and myofibril disorganisation [11, 14], however, we did not observe these features in double *dnajb6* mutants. This indicates that the muscle weakness we identified in double mutants occurs in the absence of structural damage or protein aggregation, features that have been identified as disease causing in previous DNAJB6 models. Interestingly, double mutants showed significantly more abnormal mitochondria with diffuse disorganised cristae. This is in line with recent reports of *DNAJB6* LGMD patients that showed mitochondrial abnormalities or accumulation of mitochondria [10, 30, 39].

To investigate these mitochondrial abnormalities, we first considered markers of mitochondrial damage. Double mutants only show signs of muscle weakness at adult stages, and at this timepoint we did see a significant increase in the mitophagy regulator Pink1, suggesting that mitochondria are damaged in double mutants. To investigate mitochondrial content and function, we performed citrate synthase and ATP assays using adult muscle tissue. Citrate synthase activity is known to positively correlate with mitochondrial content, cristae surface area, and respiratory state [40]. Interestingly, there was a significant decrease in citrate synthase activity in double mutants, in the absence of changes to mitochondrial size, number, or ATP levels, suggesting a reduction in cristae surface area content. This is also in line with our electron microscopy results where we observed a significant increase in diffuse mitochondrial cristae in double mutants. Whilst we did not observe changes to ATP levels, it is possible that a reduction in ATP would be observed at later timepoints. A reduction in citrate synthase activity can lead to impaired glucose tolerance and lipid metabolism [41]. In our model, a decrease in citrate synthase activity, in the absence of changes to ATP levels, likely indicates changes in cellular metabolism, however, this requires further investigation.

Although we have not resolved how loss of *dnajb6* leads to mitochondrial abnormalities, it is clear that mitochondrial changes can lead to skeletal myopathies. Mutations which affect key mitochondrial components like ATP synthase subunits [42, 43], dehydrogenases [44, 45] or mitochondrial tRNA synthetases [46, 47], are known as mitochondrial myopathies and whilst these can have multisystemic effects, muscle weakness is a common finding [48]. Future studies should consider different aspects of mitochondrial function in double mutants in order to determine how Dnajb6 interacts with mitochondrial pathways. These additional studies will create a clearer picture around the role of Dnajb6 in the muscle and how loss of *dnajb6* leads to mitochondrial abnormalities and muscle weakness.

Previously, the unregulated interaction between DNAJB6 and Hsp70 leading to defective protein quality control was identified as the cause of myopathic changes [13]. However, we show here that *dnajb6* mutants develop muscle weakness independently of defects in protein homeostasis. Our work demonstrates that loss of Dnajb6 leads to mitochondrial abnormalities. This is consistent with the description of mitochondrial damage in recessive [10] and dominant cases [30] of *DNAJB6* myopathy. In addition, our results reflect findings in recessive [10] and dominant [16] *DNAJB6* mouse models which showed mitochondrial abnormalities. However, these models also showed myofibrillar damage, and so it is possible the mitochondrial defects could have been secondary to the muscle damage. In our model, the presence of reduced muscle function in the absence of evident myofibrillar damage or disruption to protein homeostasis, suggests that mitochondrial damage may contribute to muscle weakness in LGMD D1, in addition to the previously described structural and chaperone defects, and that targeting mitochondrial dysfunction may be a therapeutic strategy for LGMD D1.

## Materials and methods

### Zebrafish lines

Zebrafish were housed at the Monash University AquaCore facility and maintained according to standard procedures [49]. All adult zebrafish experiments were conducted according to ethics project ERM21188 approved by the Monash Animal Research Precinct Committee 3. Mutant lines for *dnajb6a* and *dnajb6b* were generated using CRISPR-Cas9 mutagenesis. Guide RNAs (Integrated DNA Technologies) targeting *dnajb6a* (GGAAGAGAGCACCACTTCGG) and *dnajb6b* (GTAACCCAGAAGACGTCTTC) were injected into one cell embryos using a Femtojet microinjection system (Eppendorf) at 150 ng/μl with Alt-R® S.p. HiFi Cas9 Nuclease V3 (Integrated DNA Technologies, 1081061), 300 mM KCl, 0.005% phenol red and dH_2_O to a final volume of 5 μl. Injected larvae were then raised to identify founders. Mutation sites were confirmed by flanking PCR and DNA sequencing analysis. Founders were crossed to *Tübingen* (TU) for two generations and all experiments were performed with the F_2_ generation or later.

### KASP genotyping assay

DNA was collected from larval or adult zebrafish that were anesthetised in 0.16% tricaine methane-sulfonate and cut either at the pigment gap at the end of the tail or at the edge of the caudal fin. DNA samples was extracted using the HotShot method [50]. Genotyping was completed with the allele specific KASP assay (Biosearch Technologies, KBS-2300-001). Primer sequences are listed in supplementary Table 1. PCR samples were scanned for allele specific fluorescence using a Synergy Mx microplate reader (BioTek). Genotyping analysis was completed using KlusterCaller software (Biosearch Technologies).

### Quantitative RT-PCR

Frozen muscle tissue was homogenised and extracted in TRIzol reagent at 1–6 dpf or 3 mpf (ThermoFisher Scientific, 15596018). Samples were then treated with RQ1 RNase-Free DNase (Promega, M6101) to remove any traces of DNA contamination. RNA was reverse transcribed to generate cDNA using the ProtoScript II First Strand cDNA Synthesis Kit (Genesearch, E6560L). RT-PCR was performed for 30 cycles with an annealing temperature of 58°C. Samples were run at 120 V on a 3% agarose gel and imaged with the GelDoc Go Gel Imaging System (Bio-Rad, 12009077). qRT-PCR was performed using SYBR Green I Master (Roche, 04887352001) with a Lightcycler 480 Instrument (Roche, 05015243001). Both *lsm12b* and *ef1α* were used as reference genes. Three technical replicates were used to generate an average value for each biological replicate. Melt curves for each technical replicate were analysed and those flagged by the LightCycler 480 Sw 1.5.1 software (Roche) as having multiple products (10/144 reactions) were excluded. Primers used are listed in Supplementary Table 2. Genotypes were compared using a one-way ANOVA and two-way Dunnett multiple comparisons test.

### Whole mount immunofluorescence staining

Zebrafish larvae at 1 dpf were treated with methyl cellulose as previously described [28]. Embryos were then anesthetised and fixed whole in 4% paraformaldehyde overnight at 4°C. The following day samples were washed 3 × 5 min in PBST with 0.02% Tween 20 followed by permeabilisation in acetone for 7 min at −20°C. At room temperature the samples were then washed in dH_2_O and PBST for 5 min each. Next the embryos were placed in blocking solution (2% goat serum, 1% BSA and 1% DMSO in PBST) for one hour before being incubated overnight with the primary antibody anti-Myosin (DSHB, A4.1025) diluted 1:100 in block. The following day the samples were incubated with the secondary antibody Alexa Fluor 594 (ThermoFisher Scientific, A21135). Larvae were mounted in 1% low melting temperature agarose in FEP tubing (Bohlender, S1815-04). Samples were imaged with a 20× water dipping objective with a numerical aperture of 1.0 on a Thorlabs confocal microscope (Thor Imaging Systems Division) or a 710 confocal microscope (Zeiss). Maximum intensity projections and background corrections were performed in Fiji (http://fiji.sc, [51]). Images were blinded using the random file name generator in Fiji (http://fiji.sc) and then scored for fibre damage. Data was then unblinded and genotypes were compared using a binomial test in SPSS statistics (IBM). Immunofluorescence analysis was completed with seven biological replicates.

### Locomotion assays

Functional assessment of zebrafish at 6 dpf was performed with the Zebrabox (Viewpoint) as previously described [52]. The researcher was blinded to zebrafish genotype and fish were randomly arranged based on a random number generator. Video recordings of zebrafish were analysed using Ethovision software (Noldus, version 14). Analysis was completed with three replicates and genotypes were compared with a linear model in SPSS statistics (IBM).

Long term swimming analysis was completed with zebrafish genotyped at 5 dpf. Adult swimming experiments were performed weekly up until 3 mpf. The experiment was completed at the same time, day and location, each week in order to minimise extraneous variables. Zebrafish were placed into individual tanks and then tanks were arranged in a randomised pattern according to a random number generator. Each set of tanks was then placed into the ZebraCube (Viewpoint) and given time to acclimatise to the environment. Fish were recorded for a period of 10 min. Video recordings were then analysed blinded using Ethovision software (Noldus, version 14). This assay was completed blinded with six groups per genotype across 3 biological replicates. The mean swimming distance of each group was used to compare genotypes with a mixed linear model in SPSS statistics (IBM), where replicate was a random factor and genotype was a fixed factor.

### Survival

Long term survival of genotyped larvae was monitored from 6 dpf until 12 wpf. A maximum of 16 zebrafish were raised per genotype per tank. Fish were monitored daily for signs of health issues and deaths were recorded for each animal. Fish displaying clinical signs of ill-health or distress were euthanised with tricaine methane-sulfonate at a concentration of 300 mg/l or greater. Kaplan–Meier survival curves were compared using the Mantel-Cox log rank test in SPSS statistics (IBM).

### Western blotting

Prior to protein extraction, larvae being assessed for autophagic flux were treated with 40 μM of NH4Cl or dH_2_O for 16 h. Zebrafish larval or adult muscle tissue samples were homogenised in 100 μl of RIPA buffer (50 mM Tris–HCl pH 7.4; 150 mM NaCl, 22 mM EDTA; 1% NP-40, 0.1% SDS), with protease inhibitor cocktail (Roche, 11836153001) via sonification (Branson Ultrasonics). Then 25 μl 5× LSB (62.5 mM Tris–HCl pH 6.8; 10% glycerol, 0.01% bromophenol blue; 2% SDS) was immediately added to solubilise and denature proteins. Each sample was reduced by adding 2 μl of 10× DTT (Sigma-Aldrich, 646563) to 18 μl of protein extract and then heating at 100°C for 10 min. Samples were loaded into NuPAGE™ 4 to 12%, Bis-Tris gels (ThermoFisher Scientific, NP0335BOX) and run at 150 V for 1 h. Following separation protein samples were transferred to a hydrophobic PVDF membrane (Sigma-Aldrich, IPVH00005). Membranes were blocked in 5% milk PBST for 1 h at room temperature. Samples were then probed with primary antibodies diluted in 5% milk PBST overnight at 4°C. Primary antibodies used were anti-p62 (Cell Signalling Technology, 5114S), anti-LC3A/B (Cell Signalling Technology, 12741), anti-Pink1 (Cell Signalling Technology, 6946) and anti-VDAC (Abcam, ab154856). The following day, membranes were probed with a HRP-conjugated secondary antibody (Southern Biotech, 4010-05, 1010-05) diluted in 5% milk PBST and then stained with Amersham™ ECL™ Prime Western blotting detection reagent (Bio-Strategy, GEHERPN2232). Chemiluminescence was imaged using a GelDoc Go Gel Imaging System (Bio-Rad, 12009077). Membranes were then stripped, blocked and probed with anti-GAPDH (Santa-Cruz Biotechnology, sc-47724) using the same procedure as above. Western blots were quantified using ImageJ software and GAPDH was used as a loading control. Autophagic flux was determined by subtracting untreated LC3-II levels from NH_4_Cl treated LC3-II levels. Values were adjusted according to GAPDH levels and then normalised to the average of all values within the replicate, to account for differences between replicates. Western blot analysis at 6 dpf was performed with three replicates and analysis at 3 mpf was performed with samples from eight adult fish per genotype across three biologcial replicates. Statistical significance was assessed using a one-way ANOVA followed by a two-way Dunnet multiple comparisons test in SPSS statistics (IBM).

### Electron microscopy

Tissue samples from larval or adult zebrafish were placed into primary fixative (2.5% glutaraldehyde, 2% paraformaldehyde in 0.1 M sodium cacodylate buffer), overnight at 4°C. The tissues were then rinsed in fresh sodium cacodylate buffer for 3 × 15 min. Secondary fixation was performed using 1% osmium tetroxide and 1.5% potassium ferricyanide in cacodylate buffer for 1 h at room temperature. The tissues were then washed in dH_2_O for 3 × 15 min. Fixed tissues were dehydrated with increasing concentrations of ethanol for 15 min, consisting of 30, 50, 70, 90 and 100% ethanol. The ethanol was removed and replaced with 100% propylene oxide. Dehydrated tissues were incubated in a mixture of Epon resin and propylene oxide at a ratio of 1:1 for 6 h at RT, followed by a 2:1 Epon/propylene oxide mixture overnight. Tissues were incubated in 100% freshly made Epon resin for 6 h, followed by another 100% resin change overnight. The tissues were then placed into Beem capsules in 100% resin and the resin polymerised for 48 h in an oven at 60°C.

Resin embedded tissue was sectioned with a Diatome diamond knife using a Leica UCS ultramicrotome. Sections of thickness 70—90 nm were collected onto 150 mesh copper grids and stained sequentially with 1% uranyl acetate for 10 min and lead citrate for 5 min. The sections were imaged in a JEOL 1400+ transmission electron microscope at 80 kV, and images captured with a digital camera at a resolution of 2 K × 2 K. Images were taken from random areas of the muscle by choosing the first sub-sarcolemma region of a muscle fibre observed in each consecutive grid square of the TEM grid, scanning across the grid in a raster pattern.

Each image file was assigned a blinded file name to remove bias during analysis. The blinded images were then manually assessed for mitochondrial abnormalities. Quantification of mitochondrial abnormalities was completed with two biological replicates and four different zebrafish samples per genotype. A binomial test was used to test for differences between genotypes in abnormal mitochondria accumulation using SPSS statistics (IBM).

### Metabolic assays

Frozen adult muscle tissue samples were homogenised using a Bead Ruptor Homogeniser (Omni International) and prepared according to the manufacturer’s instructions for citrate synthase (Abcam, ab239712) and ATP assays (Abcam, ab83355). For each assay a standard curve was calculated using provided standards to determine sample concentration. For both assays the samples were plated and measured for colorimetric absorbance at 25°C. The concentration of each sample was normalised to the amount of tissue used in mg. Analysis was completed with five samples from three biological replicates. To examine differences in genotypes a one-way ANOVA was used with a two-way Dunnett multiple comparisons test in SPSS statistics (IBM).

### Statistical analysis

Statistical tests used for each dataset are described above and in relevant figure legends. All statistical analysis was performed using SPSS statistics (IBM) and graphs were generated with Prism 10 (GraphPad Software). Data is presented as the mean ± SEM and a *P* value of < 0.05 was considered statistically significant.

## Supplementary Material

SupplmentaryData_ddae061

ARRIVE_McKaigeetal_ddae061

## Data Availability

Experimental data files and statistical analysis are available at https://bridges.monash.edu/projects/Mitochondrial_abnormalities_contribute_to_muscle_weakness_in_a_DNAJB6_deficient_dystrophy_zebrafish_model/173751.
