# Supplementary material for: Mitochondrial abnormalities contribute to muscle weakness in a Dnajb6 deficient zebrafish model
Source: Hum Mol Genet. 2024 Apr 15;33(14):1195–206. doi: 10.1093/hmg/ddae061 (PMC11227618; doi:10.1093/hmg/ddae061)
Supplement: SupplmentaryData_ddae061 [file supplmentarydata_ddae061.pdf]

Mitochondrial abnormalities contribute to muscle weakness in a *DNAJB6* deficient model.

Emily A. McKaige[1], Clara Lee[1], Vanessa Calcinotto[1], Saveen Giri[2], Simon Crawford[3], Meagan J. McGrath[2], Georg Ramm[2,3], Robert J. Bryson-Richardson[1*]

[1]School of Biological Sciences, Monash University, Melbourne, Australia

[2]Department of Biochemistry and Molecular Biology, Biomedicine Discovery Institute, Monash University, Clayton, Victoria 3800, Australia

[3]Monash Ramaciotti Centre for Cryo Electron Microscopy, Monash University, Clayton, Victoria 3800, Australia.

*Corresponding author: Robert J. Bryson-Richardson

Email: robert.bryson-richardson@monash.edu

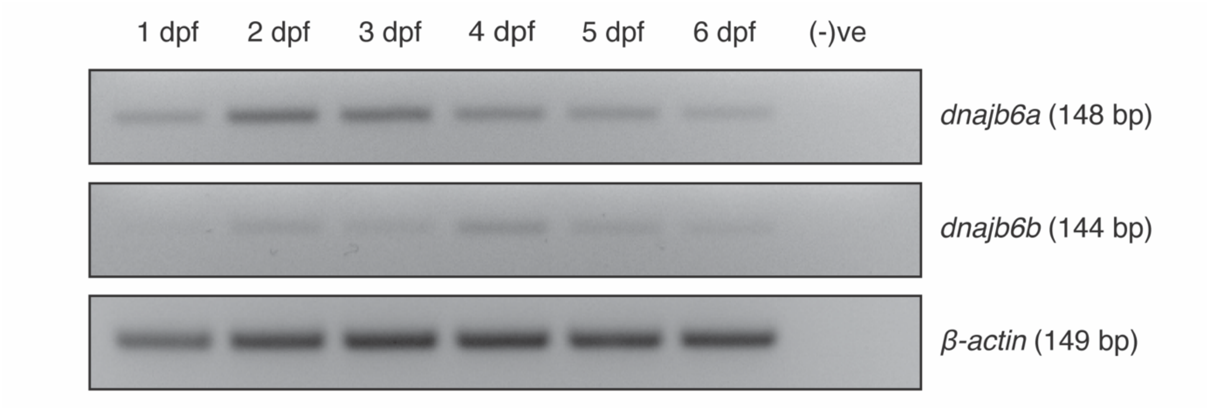

Supplementary Figure 1. Expression of *dnajb6a* and *dnajb6b* during early development.

β-actin was used as a loading control. Negative control contains H_2O instead of DNA.

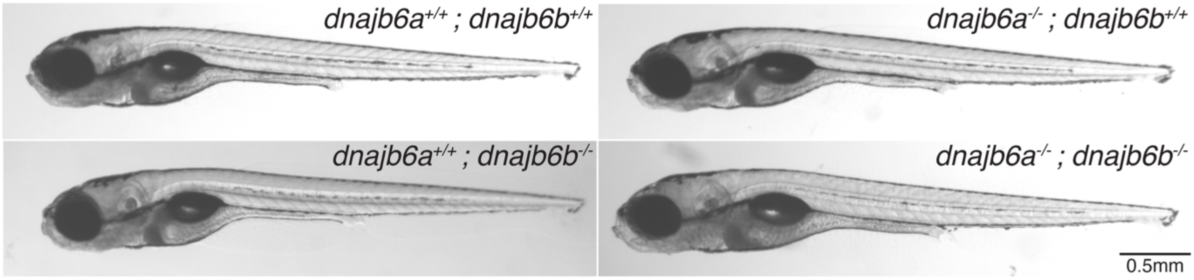

Supplementary Figure 2. Normal appearance of *dnajb6* larvae at 6 dpf

Brightfield images of larvae show no phenotypic differences between *dnajb6* mutants and wildtype.

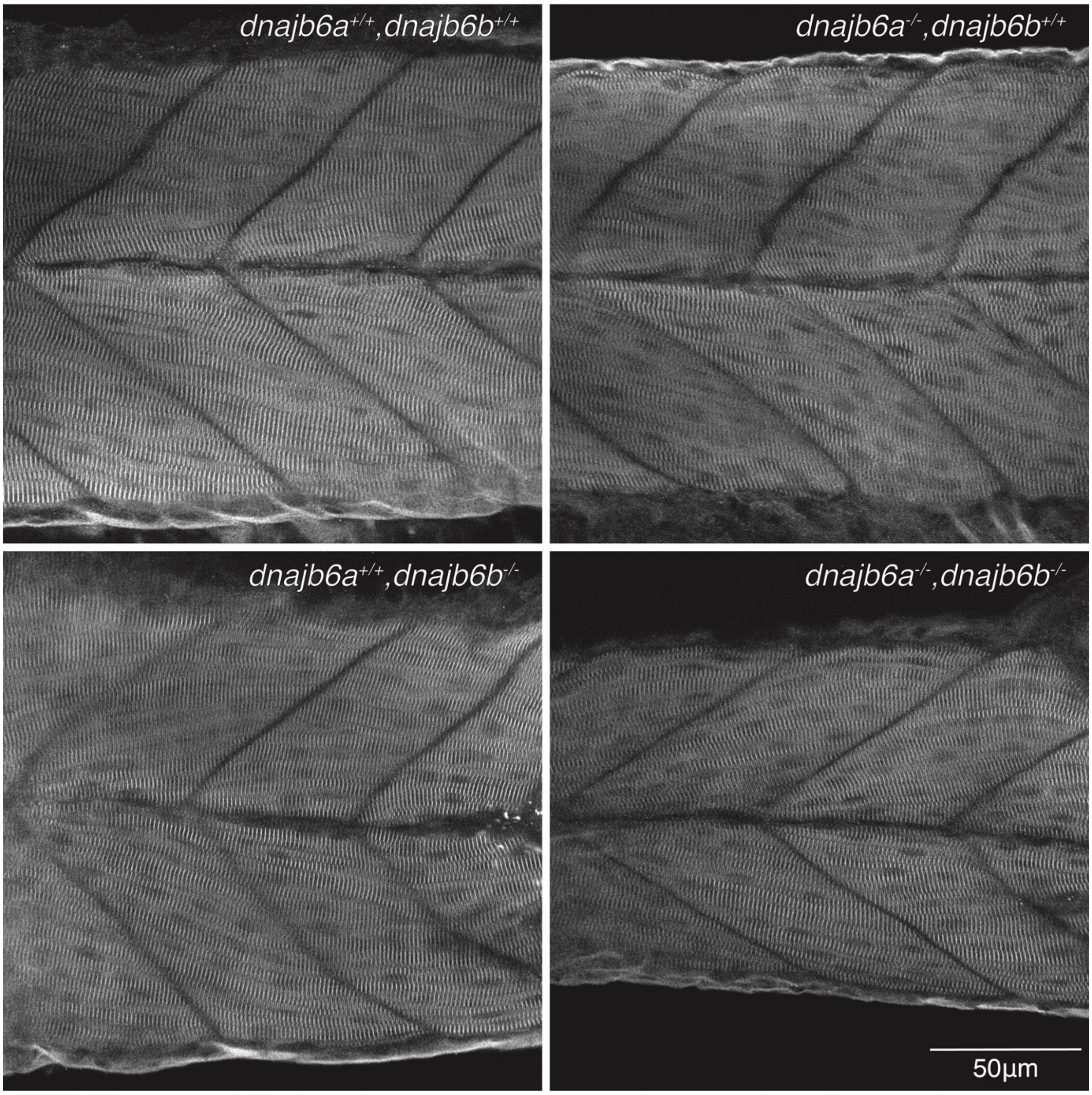

Supplementary Figure 3. Fibre patterning is not affected in *dnajb6* mutants at 2 dpf.

Analysis of the Z-disk reveals no significant differences between *dnajb6* mutants and siblings. Larvae were labelled with anti-α-actinin and imaged laterally. Maximum projections were obtained from confocal z series.

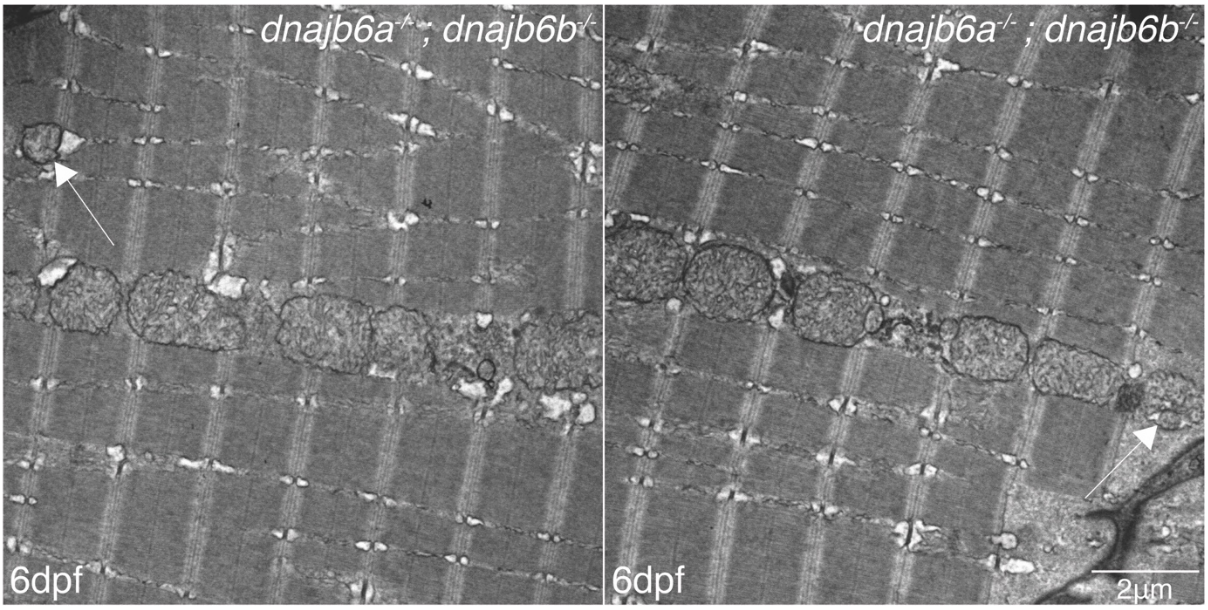

Supplementary Figure 4. Representative images of abnormal mitochondria in double *dnajb6* mutants at 6 dpf.

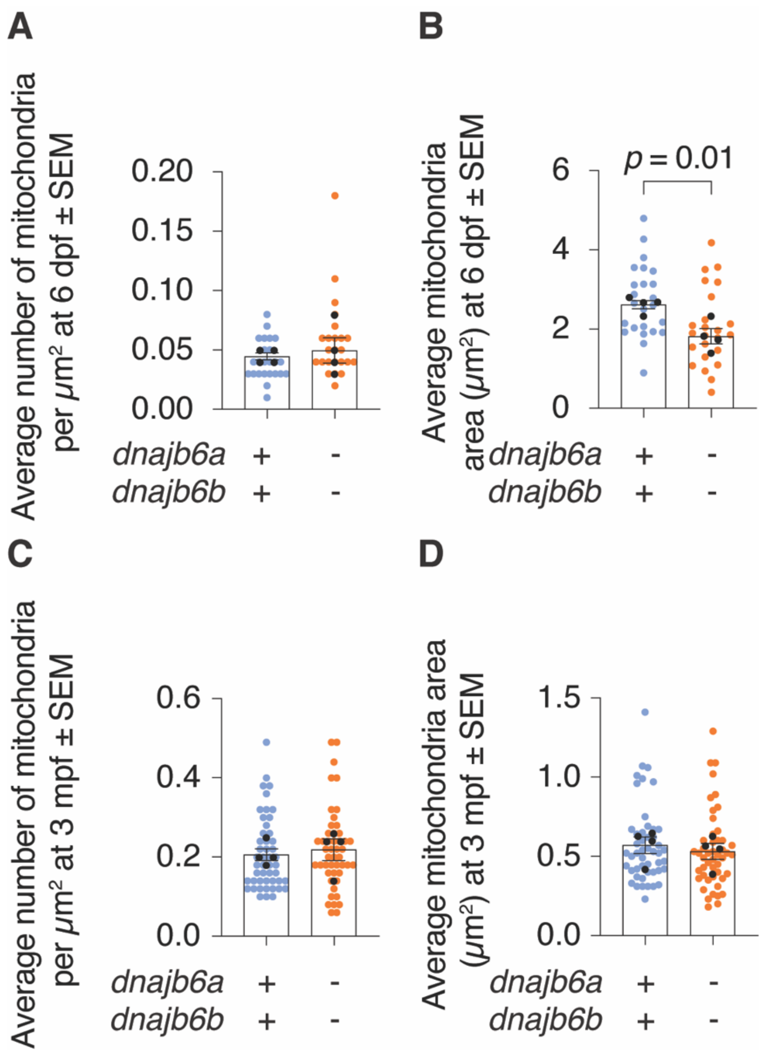

Supplementary Figure 5. Quantification of mitochondria in electron microscopy images.

(A) At 6 dpf the average number of mitochondria was not significantly different between double mutants and wildtype. **(B)** The average mitochondria size was significantly decreased in double mutants compared to wildtype at 6 dpf. **(C-D)** Mitochondria number and size in double mutants was not significantly different to wildtype siblings at 3 mpf. Genotypes were compared using a Students t- test. Analysis was completed using averaged data from individual fish (black dots). Results from individual electron microscopy images are displayed in coloured dots. Analysis was completed blinded with four individual fish and two biological replicates.

Supplementary Table 1. KASP allele specific primer targets.

Gene	Primer Sequence
dnajb6a	5’-AATTTGTCACTTTTCTTCAATGTTTGGACAGGAGGAGGAAGAGAGCACCA[CTTC/]GGAGGAGGAGGATTCACATTCCGCAATCCAGAGGACGTTTTC AGGGAATT-3’
dnajb6b	5’-TACGATGATGAATATATGGGTGGATTCACATTCCGTAACCCAGAAGACGT[CT/]TCAGGGAATTTTTTGGAGGTCATGATCCATTTGCAGATTTCTTTGGTAGG-3’

Supplementary Table 2. Primers sequences used for qRT-PCR and RT-PCR analyses.

Gene	Analysis	Primer Sequence	Orientation
dnajb6a	Fig. 1	5’-ATGGCATCCAGATAAGAACCCG-3’	forward
dnajb6a	Fig. 1	5’-TCCTCCTGGTGTTAAGCCTTC-3’	reverse
dnajb6b	Fig. 1	5’-CCTTGGTGTCACGAAAAGCG-3’	forward
dnajb6b	Fig. 1	5’-TCTGACAGGACTTCATATGCTTCT-3’	reverse
ef1α	Fig. 1	5’-CCATTGACATTGCTCTCTGGAA-3’	forward
ef1α	Fig. 1	5’-AACACCACCAGCAACAATCAG-3’	reverse
lsm12b	Fig. 1	5’-AGTTGTCCCAAGCCTATGCAA-3’	forward
lsm12b	Fig. 1	5’-ACCTGGTACGGTGGTGAGAT-3’	reverse
dnajb6a	Supp Fig. 1	5’-GCAGCCATAGAAGTTGGTGGA-3’	forward
dnajb6a	Supp Fig. 1	5’-TGCATCTTCCTTATTATCCGGGTT-3’	reverse
dnajb6b	Supp Fig. 1	5’-GCACTAAAATGGCATCCAGACA-3’	forward
dnajb6b	Supp Fig. 1	5’-AGAGAGGCCTTGTTTACCATATCT-3’	reverse
β-actin	Supp Fig. 1	5’-GCATTGCTGACCGTATGCAG-3’	forward
β-actin	Supp Fig. 1	5’-GATCCACATCTGCTGGAAGGTGG-3’	reverse
